# Numerical Simulation of Free Surface Deformation and Melt Stirring in Induction Melting Using ALE and Level Set Methods

**DOI:** 10.3390/ma18010199

**Published:** 2025-01-05

**Authors:** Pablo Garcia-Michelena, Emilio Ruiz-Reina, Olaia Gordo-Burgoa, Nuria Herrero-Dorca, Xabier Chamorro

**Affiliations:** 1 Mechanical and Manufacturing Department, Mondragon University, 20500 Mondragon, Spain; ogordo@mondragon.edu (O.G.-B.); nherrero@mondragon.edu (N.H.-D.); xchamorro@mondragon.edu (X.C.); 2 Institute Carlos I for Theoretical and Computational Physics (iC1), Department of Applied Physics II, University of Malaga, 29071 Malaga, Spain; eruizr@uma.es

**Keywords:** induction melting, multiphysics modeling, free surface, electromagnetic stirring

## Abstract

This study investigates fixed and moving mesh methodologies for modeling liquid metal–free surface deformation during the induction melting process. The numerical method employs robust coupling of magnetic fields with the hydrodynamics of the turbulent stirring of liquid metal. Free surface tracking is implemented using the fixed mesh level set (LS) and the moving mesh arbitrary Lagrangian–Eulerian (ALE) formulation. The model’s geometry and operating parameters are designed to replicate a semi-industrial induction melting furnace. Six case studies are analyzed under varying melt masses and coil power levels, with validation performed by comparing experimentally measured free surface profiles and magnetic field distributions. The melt’s stirring velocity and recirculation patterns are also examined. The comparative analysis determines an improved performance of the ALE method, convergence, and computational efficiency. Experimental validation confirms that the ALE method reproduces the free surface shape more precisely, avoiding unrealistic topological changes observed in LS simulations. The ALE method faces numerical convergence difficulties for high-power and low-mass filling cases due to mesh element distortion. The proposed ALE-based simulation procedure is a potential numerical optimization tool for enhancing induction melting processes, offering scalable and robust solutions for industrial applications.

## 1. Introduction

Electromagnetic metallurgy encompasses technologies such as VIM and ISM [1], levitation melting [2], or continuous casting [3]. It deals with physical phenomena such as magnetic fields, fluid dynamics, heat transfer, and their coupling and interaction [4]. In induction melting, the alternate current in the coil generates a magnetic field, inducing currents in the fluid and creating a secondary magnetic field that distorts the original. At the liquid metal boundary, Lorentz force deforms the free surface, driving flow recirculation or stirring [5]. A stable free surface ensures efficient mixing and uniform heating, while instability affects process constancy and control [6]. Accurate free surface simulations are crucial for predicting flow stirring behavior, velocities, and heat transfer, making numerical models essential for understanding and enhancing induction melting.

Free surface profile simulation involves a multiphase flow composed of liquid metal and surrounding air. Initial approaches to calculating free surface deformation relied on steady-state analytical models balancing Lorentz force and hydrostatic pressure [7]. These hydrostatic models only accounted for the potential component of the Lorentz force, neglecting the rotational component associated with metal stirring [8].

Kirpo developed a finite element method (FEM) model based on 2D calculations of the free surface, incorporating flow contribution. The author assumed that the moving fluid does not influence the external magnetic field, as the flow operates under a low magnetic Reynolds number flow, resulting in a diffusive magnetic field distribution. Consequently, the simulation was divided into electromagnetic and fluid dynamic components, solved sequentially with a weak numerical coupling. The magnetic field was first solved to determine the Lorentz force, acting as a mechanical momentum input for the flow field. Then, the steady-state hydrodynamic flow was computed using the k−ε turbulence model and the volume of fluid (VOF) method to track the free surface deformation. However, the model was limited to small deformation cases [9].

Spitans emphasized the necessity of including the hydrodynamic component for more precise modeling [10]. In decoupled simulations, a short time interval was assumed to allow for geometry modifications during each flow computation step, treated as a constant Lorentz force distribution. Data transfer between ANSYS CFX^®^ and ANSYS Classic^®^ was facilitated by an external coupler, which transferred free surface coordinates and connectivity, formed elementary polygons, and filtered distorted surface elements [11]. The hydrodynamic model was solved using the finite volume method (FVM), incorporating turbulence effects with the k−ω SST model, while the magnetic field was computed using the FEM.

Bojarevics and Pericleous [12] developed a model using a continuous coordinate transformation method to map a deformed boundary onto a sphere. The electromagnetic field was computed using a volume integral method, while equations for the flow field were initially expressed in spherical coordinates and then transformed into new coordinates with appropriate derivative expressions. Turbulence effects were accounted for using the generalized mixing length model [13]. These transformed equations were solved numerically using a spectral collocation method. A key drawback of these methods lies in the complexity of the coordinate transformation, and spectral methods are constrained when dealing with complex boundary conditions [14].

Currently, no consensus exists on a preferable RANS turbulence model for induction melting. Bulínski et al. [15] compared RNG k−ε, k−ω, and k−ω SST models under identical induction melting conditions and operating parameters. All three models predicted nearly identical free surface curvatures, determining that the Lorentz force was the dominant variable. The authors concluded that the RNG k−ε model is the most accurate in reproducing the stirring effect, yielding lower velocity predictions.

Typically, free surface measurements have relied on contact-based techniques to determine the maximum height or the overall profile of the free surface [16]. Regarding non-contact measurement methods, Bansal et al. [17] used structured light projection to study the temporal evolution of free surface deformation. This method involved projecting a band pattern onto the surface, with any deformation altering the pattern captured by the camera. However, this approach also encountered limitations near the crucible wall due to shadowing effects, leaving this region unmeasured. Similarly, a 3D laser followed by triangulation through camera-captured reflections was used to capture free surface deformation. The results revealed a plain area in the near-wall region caused by metal oxides or slag accumulation, which distorted the surface profile. Even though the profile shape was successfully reproduced, an underestimation of the height was observed near the wall region due to the limitations of the laser and uncertainty [15].

All these above-mentioned studies employed fixed mesh or Eulerian methods, such as the volume-of-fluid (VOF) approach, for free surface tracking. Eulerian methods are popular due to their volume conservation and ability to handle complex deformations [18]. However, the reliability of the results depends on mesh resolution and numerical diffusion in the interphase, where the surface tension is introduced as volume forces [19].

In contrast, moving mesh or Lagrangian methods provide high accuracy for free surface flows with surface tension by defining computational points on free surface boundaries. These methods explicitly track surface deformations, incorporate surface tension as stress, reduce dependency on spatial resolution, and allow for accurate solutions with lower computation resource requirements [20]. However, they are limited to moderate free surface deformations with a constant topology [21].

Therefore, the present study proposes a numerically efficient and holistic simulation method for induction melting, incorporating free surface deformation dynamics and melt stirring. Strong numerical coupling between magnetic fields and fluid dynamics is implemented, including turbulence effects modeled using RANS models. Free surface tracking is performed using the level set (LS) method for fixed mesh and the arbitrary Lagrangian–Eulerian (ALE) method for moving mesh. Semi-industrial scale furnace experiments provide the data for validation, and numerical results are compared against magnetic field and free surface measurements to assess the accuracy and applicability of both approaches.

## 2. Numerical Model

### 2.1. Geometry and Material Parameters

**Figure 1:**
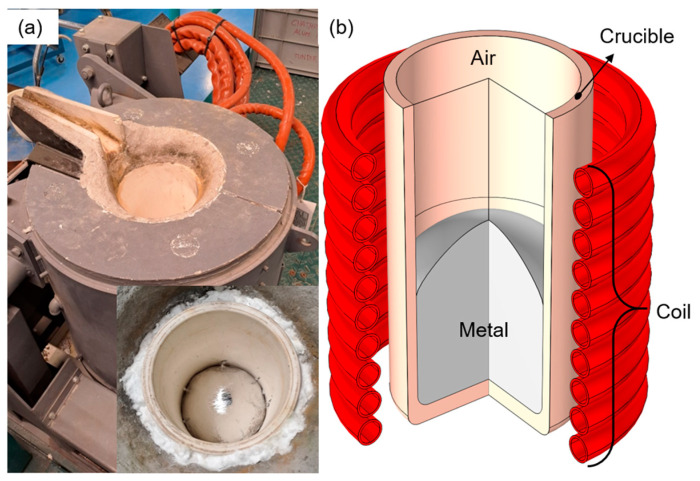
(**a**) Semi-industrial induction melting furnace. (**b**) Depiction of model geometry and domains.

As a reference for the numerical modeling and posterior experimental validation, an Inductotherm^®^ Small Steel Shell (Ondarlan S.L., Errenteria, Spain) induction melting furnace with a VIP Power Track+^®^ power generator is employed (Figure 1a). The inductor copper coil has 10 coaxial turns with water refrigeration. Electrical operational variables are equivalent to industrial capacities, having a rated power of 50 kW at 3 kHz, and the crucible has a capacity of 7.5 dm^3^ or 20 kg of aluminum. The coil turns are protected with refractory concrete placed between the crucible and coil; it is paramagnetic and has not been considered in the model. The model geometry is depicted in Figure 1b, and the detailed dimensions are shown in Table 1, including the coil, crucible, liquid metal placed inside, and the remaining air.

Also, the model material parameters and principal variables are summarized in Table 1. Pure liquid aluminum properties have been considered [22]. The properties of the rest of the material—copper for the coil, and alumina for the crucible and surrounding air—were obtained from the COMSOL Multiphysics^®^ (COMSOL AB., Stockholm, Sweden) ndatabase. The electrical conductivity of copper is 6 × 10^7^ (S/m), while the air and the crucible are electrical insulators, and the magnetic permeability of all materials has been considered equal to free space; thus, the relative permeability $\mu_{r}=1.$

### 2.2. Assumptions

The coupled magnetic field and fluid dynamics model determine the free surface and stirring. Due to the different time scales, the magnetic field is modeled in the frequency domain, while the fluid flow is obtained using a time-dependent calculation [23].

An air domain surrounds the coil, crucible, and melt to expand the magnetic field, including a fair field domain for replicating an infinite domain. The revolution symmetry of the geometry is exploited by adding an axisymmetric axis. This approach reduces the dimensionality of the domains that need to be discretized, considering them in two dimensions. It assumes that the magnetic vector potential has a component only in the axial direction and that the electric potential varies only in the vertical direction, disregarding the coil helicity and the sinusoidal shape of the inductor [24]. The time-averaged Lorentz force is the source term for the fluid flow.

The metal is always in a liquid state, incompressible, and Newtonian. The temperature effects are not considered; thus, natural convection due to buoyancy forces and Marangoni effects are neglected, which are negligible compared to electromagnetic forces. The Reynolds number of the induction-melting falls in the order of 10^4^–10^5^; thus, RANS turbulence models are considered efficient in capturing turbulence and appropriate for reproducing the averaged flow [25].

### 2.3. Governing Equations

#### 2.3.1. Magnetic Field Submodel

Maxwell’s equations are employed for the resolution of the magnetic fields, assuming that the power generator feeds the coil with an alternating harmonic electric current $I_{c}=I_{0}\sin\left(\omega_{f}t\right)$. Consistently, the equations are expressed in the frequency domain by applying a pure imaginary exponential dependence $\exp\left({j\omega }_{f}t\right)$. Assuming no accumulation of electric charge and no free currents, the partial differential equation (PDE) of the time-harmonic Maxwell–Ampère law for the magnetic field is written as

(1) $$\nabla \times H=J+j\omega_{f}\varepsilon_{0}E$$

where H is the magnetic field strength, A/m; J is the current density, A/m^2^; $\varepsilon_{0}$ is the electrical permittivity in vacuum 8.85 × 10^−12^ (F/m); and E is the electric field, V/m. We now consider Ohm’s law and the interaction between the magnetic field and the motion of the conducting liquid metal medium by the Lorentz force J=σ(E+u×B), where B is the magnetic flux density, T; σ is the electrical conductivity, S/m, and u is the convective motion of the melt velocity, m/s. The current density is generalized by introducing an externally generated current ($J_{e})$, and the following expression is derived:

(2) $$\nabla \times H=\sigma \left(E+u\times B\right)+j\omega_{f}\varepsilon E+J_{e}$$

The magnetic vector potential **A**, T·m, is necessary for the resolution of the fields according to the **A** formulation, satisfying the magnetic Gauss law ∇ · **B** = 0, as ∇ · (∇ × **A**) = 0 always holds. Thus, we can use B=∇×A, and the electric field from $E=-j\omega_{f}A$, along with the constitutive relation $B=\mu_{r}\mu_{0}H$. The time-harmonic Maxwell–Ampère law equation can be finally rewritten as

(3) $$\nabla \times \left(\mu_{r}^{-1}\mu_{0}^{-1}\nabla \times A\right)+j\omega_{f}\sigma A=J_{e}+\sigma (u\times \left(\nabla \times A\right))$$

where $\mu_{0}$ is the permeability of the vacuum, 4π × 10^−7^ (H/m). The left side represents the diffusion and the time-harmonic behavior of the magnetic vector potential A, while the right side accounts for the induced current due to the metal flow in the magnetic field.

The external current density $J_{e}$ is determined by the out-of-plane component of the current ($I_{C}$) passing through the coil, A. The coil is modeled as a single conductor, essentially a solid region of copper through which the current flows. By constraining the total integrated current of $I_{C}$, the electric potential $V_{c}$, V, can be estimated.

(4) $$J_{e}=\sigma V_{c}/L$$

where *L* represents the coil length of 2π*r*, m, and *r* is the coil radius, m. Power input $P_{C}$, kW, introduces non-linearity in the equations, necessitating the specification of the phase angle between the two vectors. Thus, power excitation is imposed on the coil, introducing the following additional constraint:

(5) $$P_{C}=\frac{1}{2}Re\left(V_{C}{\cdot I}_{C}^{*}\cdot cos(\varphi )\right)$$

#### 2.3.2. Fluid Dynamics Submodel

The flow transport due to the magnetic field compromises the consideration of free surface hydrodynamics, metal flow transport, and turbulence, as governed by the Navier–Stokes equations for mass and momentum conservation over time:

(6) ∇·(ρu)=0

(7) $$\rho \frac{\partial u}{\partial t}+\rho \left(u\cdot \nabla \right)u=\nabla \cdot \left[-pI+K\right]+F$$

where ρ is the fluid density, kg/m^3^; u is the fluid velocity, m/s; p is the pressure, N/m^2^; I is the identity matrix; K is the viscous stress tensor, given by $K=(\mu +\mu_{T})(\nabla u+{\left(\nabla u\right)}^{T})$, N/m^2^, which describes the fluid’s internal resistance and friction forces; and F represents the applied volume forces, N/m^3^.

To account for the flow turbulence and consequent turbulent viscosity $\left(\mu_{T}\right)$, kg/(m·s), the standard k−ε model is adopted with wall function approximation to compute the sub-viscous layer velocity, whose formulation can be found elsewhere [26]. This model introduces two additional transport equations for two dependent variables: the turbulent kinetic energy (k), m^2^/s^2^, and the dissipation rate (ε), m^2^/s^3^:

(8) $$\rho \frac{\partial k}{\partial t}+\rho u\cdot \nabla k=\nabla \cdot \left(\left(\mu +\frac{\mu_{T}}{\sigma_{k}}\right)\nabla k\right)+P_{k}-\rho \varepsilon$$

(9) $$\rho \frac{\partial \varepsilon }{\partial t}+\rho u\cdot \nabla \varepsilon =\nabla \cdot \left(\left(\mu +\frac{\mu_{T}}{\sigma_{\varepsilon }}\right)\nabla \varepsilon \right)+C_{\varepsilon 1}\frac{\varepsilon }{k}P_{k}-C_{\varepsilon 2}\rho \frac{\varepsilon^{2}}{k}$$

(10) $$\mu_{T}=\rho C_{\mu }\frac{k^{2}}{\varepsilon }$$

where μ is the dynamic viscosity, kg/(m·s); the turbulent production rate $P_{k}=\mu_{T}\left(\left(\nabla u:\left(\nabla u+\nabla u^{T}\right)\right)\right)$; and the constants of k−ε formulation are $\sigma_{k}=1.0$, $\sigma_{\varepsilon }=1.3$, $C_{\varepsilon 1}=1.44$, $C_{\varepsilon 2}=1.92,$ and $C_{\mu }=0.09$ [26].

Among the external forces (F), the predominant force acting on the free surface and flow stirring is the magnetic pressure generated by the Lorentz force ($F_{L}$), N/m^3^. For a harmonic field, the time-averaged component of the Lorentz force is

(11) $$F_{L}=\frac{1}{2}Re\left(J\times B^{*}\right)$$

where Re denotes the real part and $B^{*}$ is the complex conjugate of B. With this term, magnetic and fluid dynamic field coupling is accomplished.

The calculation of the hydrostatic force is straightforward, as it is the volume force generated by gravity (ρg). Finally, the surface tension force ($F_{ST}$) depends on the modeling procedure for the multiphase problem and the corresponding interface tracking method. The equilibrium of these forces results in free surface and a final stationary surface profile.

#### 2.3.3. Multiphase Flow Submodel

After all the forces acting on the liquid metal are determined, an interface tracking method is necessary to model the free surface displacement and the interaction between the two phases. Two methods are compared: fixed mesh and moving mesh. The level set method (LS) is chosen for the fixed mesh approach, while the arbitrary Lagrangian–Eulerian (ALE) method is selected for the moving mesh approach.

(a) LS Method

The evolution of the interface over time is tracked by solving the advection equation for the LS function, which is the volume fraction of phase $\phi \in \left[0,1\right]$. Thus, when ϕ=1, material properties are equal to the metal liquid domain, while ϕ=0 in the air domain. Across the interphase, there is a transition from zero to one, which is the multiphase interface flow defined as the ϕ=0.5 iso-surface of the LS function. The conservative form is assigned by the integral of ϕ for exact numerical conservation. To couple the ϕ function with the Navier–Stokes equations, the interphase has to move with the flow velocity field:

(12) $$\frac{\partial \phi }{\partial t}+\nabla \cdot \left(u\phi \right)=\gamma \nabla \left(\epsilon \nabla \phi -\frac{\phi \left(1-\phi \right)\nabla \phi }{\left|\nabla \phi \right|}\right)$$

where ϕ is the LS function, γ is a reinitialization parameter (typically the expected maximum velocity), m/s, and ϵ is the interphase thickness controlling parameter, which is usually chosen as one-fourth of the maximum mesh element size, $h_{max}/4$, m. The density and viscosity are discontinuous across the interface and depend on the ϕ function using the volume average method:

(13) $$\rho =\rho_{1}+{(\rho }_{2}-\rho_{1})\phi$$

(14) $$\mu =\mu_{1}+{(\mu }_{2}-\mu_{1})\phi$$

where suffix 1 corresponds to air and suffix 2 to aluminum. For the LS method, the surface tension force $F_{ST}$, N/m^2^, acting on the interface between the two fluids is

(15) $$F_{ST}=\gamma \delta \kappa n_{i}+\delta \nabla_{S}\gamma$$

where γ is the surface tension, N/m; $n_{i}$ is the unit normal to the interface; κ=−∇·n is the curvature, 1/m; δ is a Dirac delta function located at the interface; and $\nabla_{S}$ is the surface gradient operator, $\nabla_{s}=\left(I-{n_{i}n_{i}}^{T}\right)\nabla$.

(b) ALE Method

The ALE method combines the benefits of the Eulerian and Lagrangian approaches by allowing the computational mesh to move with the fluid dynamic regions, while remaining fixed in others. This framework solves the fluid equations in a moving reference frame, with mesh motion treated as an additional variable. The process begins with a static mesh and a mapping function that relates the reference coordinates to the current mesh positions. The original mesh represents the reference frame, while the deformed mesh defines the spatial frame. In two dimensions, the deformed mesh coordinates (r, z) adjust relative to the undeformed reference coordinates (R, Z).

This approach solves the governing equations for the fluid velocity (u) and pressure (*p*) fields in the moving reference frame, incorporating mesh displacement as an additional variable. The mesh movement is directly coupled to the fluid velocity field, ensuring that the mesh nodes follow the flow dynamics by aligning their displacement with the local fluid velocities in the *R* and *Z* directions, thereby maintaining consistency between the computational domain and the physical flow field. The Winslow smoothing method ensures smooth deformation and proper propagation of domain displacement. Thus, the PDEs that describe the mesh displacement by flow velocity are

(16) $$u_{r}=\frac{\partial^{2}R}{\partial r}+\frac{\partial^{2}R}{\partial z^{2}}\;;\;u_{z}=\frac{\partial^{2}Z}{\partial^{2}r^{2}}+\frac{\partial^{2}Z}{\partial^{2}z^{2}}$$

Free mesh deformation in the normal direction is enabled at the interface between the two domains, allowing the mesh to adapt seamlessly to boundary deformations while remaining fixed in the axial axis. In the wall region, tangential mesh displacement accommodates radial movement at the triple point where the two domains and the boundary intersect. This approach ensures precise capture of free boundary deformation while facilitating tangential displacement along the crucible wall. The surface tension force $F_{ST}$ is given by the expression

(17) $$F_{ST}=\gamma \left(\nabla_{s}\cdot n_{i}\right)n_{i}-\nabla_{s}\gamma$$

### 2.4. Boundary Conditions

**Figure 2:**
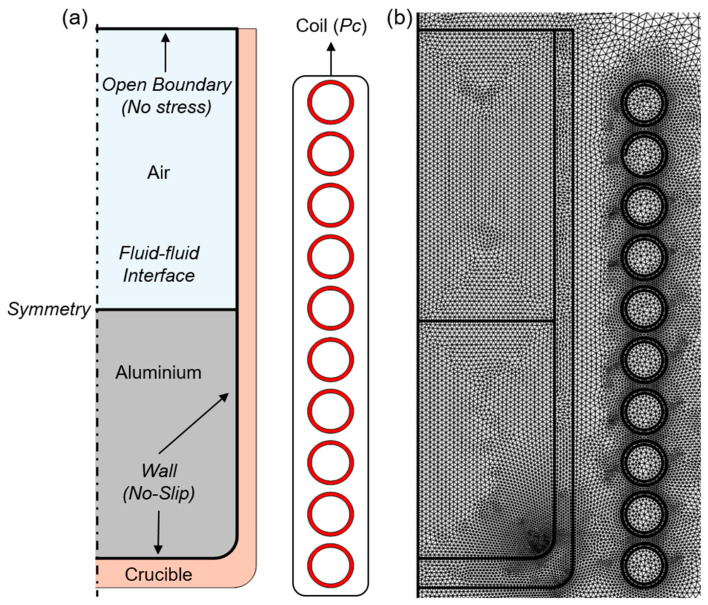
(**a**) Model domains and boundaries. (**b**) Initial mesh representation.

The boundary conditions are tailored to solve the presented equations, summarized in Table 2 and depicted in Figure 2b, including the domain and boundary assignations.

The only required boundary condition for the magnetic field is the closure of the magnetic loop with the magnetic insulation boundary condition in the fair field domain that replicates the magnetic field expansion n×A=0.

Regarding the wall, for the flow field and turbulence, a no-slip boundary condition is applied, setting u·n=0. The tangential velocity ($u_{tang})$ accounts for the fluid’s wall velocity relating to the viscous stress tensor (K). The standard wall function accounts for the turbulence effects (k and ε) in the near-wall region, based on the wall lift-off distance ($\delta_{w}^{+})$ and the von Kármán constant ($k_{v})$. The velocity in this region is approximated by a logarithmic function relating the turbulence variables based on the distance from the wall $y^{+}$, the friction velocity $u_{\tau }$ and the non-dimensional velocity $u^{+}$ [26].

The LS interface tracking method imposes a wetted wall boundary condition on the wall boundary, relating the wetting angle $\left(\theta_{w}\right)$ to the tangential velocity of the flow and the surface tension. For the ALE method, the mesh displacement in the wall is fixed in the boundary at the radial direction $d_{r}=0$, while in the radial axis, it is linked to the displacement and tangential velocity ($u_{tang}$).

A continuity condition manages mesh displacement at the fluid–fluid interface, ensuring proper coupling between the two-phase domains and accurate resolution of the flow field. With no mass transfer across the boundary, the velocities of the two phases are equal ($u_{1}=u_{2})$. The same goes for the turbulence variables and the viscous stress tensors (K) of both domains, which are related to the normal vector ($n_{i}$) at the interface, incorporating the effects of surface tension (**F_ST_**) and calculating the interface velocity, which aligns with the mesh displacement. On the contrary, for the LS method, the free surface is implicitly defined by the 0.5 of ϕ function.

The top boundary of the crucible is treated as an open boundary with no shear stress, assuming zero turbulent kinetic energy and dissipation rate in this region. The LS function is fixed to zero, corresponding to air, whereas the mesh movement at the open boundary is static to maintain mass conservation.

### 2.5. Meshing and Solver Configuration

The finite element method (FEM) implemented in COMSOL Multiphysics v6.2 discretizes the PDEs that define the numerical model. The fluid dynamic behavior of the free surface is initially simulated until it reaches a quasi-stationary state. The LS and ALE methods are applied to track each free surface interface. Following this, a complementary coupled frequency stationary study computes the fluid flow field using the final solution from the previous solution.

Mesh definition is crucial, especially when managing coupled nonlinear physical interfaces, such as flow transport and magnetic fields. The meshing process occurs in two steps, based on the simulation objectives. In the first step, triangular mesh elements with a minimum size capture the induced magnetic field caused by the skin effect. A boundary layer mesh is applied to the coil domains to accurately represent resistive losses from the self-inductance effects in the copper windings. Extremely fine triangular mesh elements cover the remaining domains, with a maximum element length of 3 mm. The same meshing approach is used for both the fixed and moving mesh methods to allow precise comparison between the numerical approaches (Figure 2b). For the fluid stirring simulation, mesh refinement enhances element quality and improves resolution, particularly at the fluid–wall interface, ensuring accurate flow computation through the viscous layer to the wall.

Second-order (P2 + P1) discretization is used for the velocity and pressure fields, while quadratic elements are employed for the magnetic vector potential. A segregated solver addresses the fields by dividing the problem into multiple steps corresponding to each physical phenomenon and solving them sequentially in each iteration. This segregated method generates smaller equation matrices but requires more iterations to achieve a convergent solution, limiting the time step to 5 ms. All fields are solved using direct solvers integrated into COMSOL Multiphysics^®^ v.6.2.

## 3. Experimental Methods

To verify the precision of the model, the free surface contour and deformation must be measured, as well as the magnetic field. Experimental trials are required, where the electrical input variables to feed the model are determined.

### 3.1. Magnetic Field Measurement

A Hirst GM08^®^ Gaussmeter was employed with an axial probe and a rated deviation of 0.5% to measure the axial magnetic field. This instrument measured the magnetic field on the crucible wall and at various points on the vertical reference, with a measurement interval of 50 mm, starting 10 mm from the bottom and extending up to 350 mm. During the experiments, the root-mean-square magnetic field of the alternating current wave was measured for three power conditions: 10 kW, 25 kW, and 40 kW. For the magnetic field measurements, three measurements were conducted for each power case at different axial points along the crucible. The uncertainty was determined using the range (min–max) method. The average value was the central point, while the extreme measurements defined the uncertainty limits. The deviation increased with higher magnetic field cases, reaching approximately 10% in the worst-case scenario.

### 3.2. Free Surface Measurement

Aluminum, with its relatively low density and high electrical conductivity, served as a melting material. In order to have a more pronounced free surface deformation, the initial metal height was kept below the height of the coils. Six free surface height profiles were measured for 5 kg, 7.5 kg, and 10 kg, and two power conditions, 25 kW and 40 kW.

**Figure 3:**
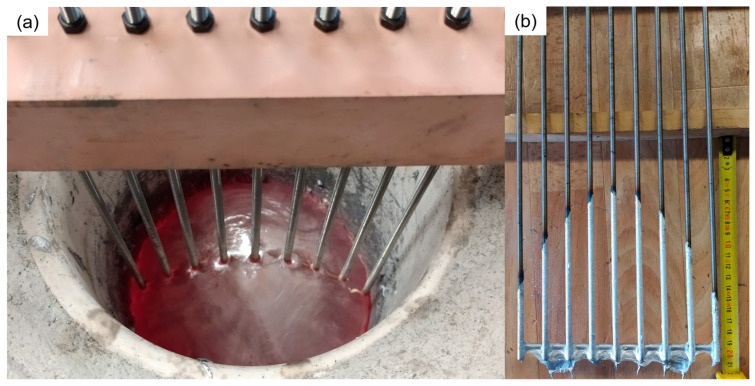
(**a**) Contact probes into the melt for free surface measurement. (**b**) Aluminum-wetted contact probes.

Aluminum ingots with a purity of 99% were melted; in each trial, the charge underwent gradual heating using stepped power stages until the charge melted entirely. Then, the temperature was elevated to 750 °C and measured using a type K thermocouple. When the free surface of the metal reached a stable state and the power input was kept constant, a device comprising nine threaded rods with a diameter of 5 mm was introduced into the metal (Figure 3a). After allowing the aluminum to wet the rods for a few seconds, the device was carefully removed, leaving a visible mark indicating the melt height and the free surface profile.

**Figure 4:**
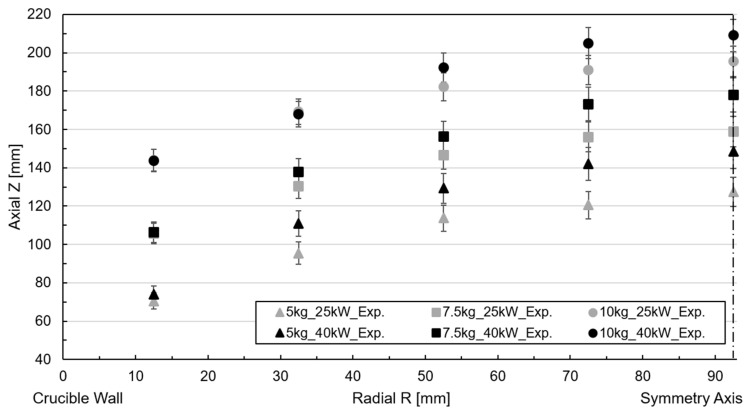
Measured free surface profile for free surface shape measurement tests.

In the contact probes, the height of the free surface was determined at various radial points by measuring the wetting height of the aluminum (Figure 3b). The average value for each radial point was used as the central point, while the maximum and minimum values defined the deviation range. Compared to the total melt height, the relative deviation ranged from 5% to 10% across all radial points plotted as error brackets in Figure 4. Notably, the deviation was minor at the center of the melt and higher near the walls. The most significant deviations were observed for the 40 kW power and 5 kg filling cases.

The characteristic convex or meniscus shape contour is consistently observed across all cases, emphasizing that the initial height of the melt varied depending on the crucible’s mass filling. For trials with lower filling levels, the melt’s height is greater, leading to a more significant difference in height between the boundary points. This trend is also observed when comparing results obtained from tests with different power supplies, with the top surface of the melt being higher for 40 kW compared to 25 kW. However, the height of the lowest reference point remains similar in both cases.

It is important to note that the contact probes in this study cannot provide precise information on the height of the metal near the crucible, thereby not being able to determine the contact between the metal and the wall accurately. However, in the melting experiments, visual inspection of the tests did not reveal significant depressions or deviations in the free surface near the wall. Instead, the free surface profile appeared continuous (Figure 3a), similar to extending the experimental measurements with an interpolation function up to the crucible wall. The measured electrical variables of the power unit will serve as input for the simulation, as summarized in Table 3.

## 4. Results and Discussion

The verification and correlation of the model were done in two parts. First, a numerical coupling and mesh independence analysis assessed the numerical dispersion of the results and ensured repeatability. Second, the model’s accuracy was evaluated by comparing the axial magnetic field and free surface profile with experimental references.

### 4.1. Numerical Verification

The robustness and reliability of the numerical model were verified, ensuring the strong numerical coupling of the induction melting physics and the mesh’s independence concerning the mesh resolution.

#### 4.1.1. Simulation Coupling

**Figure 5:**
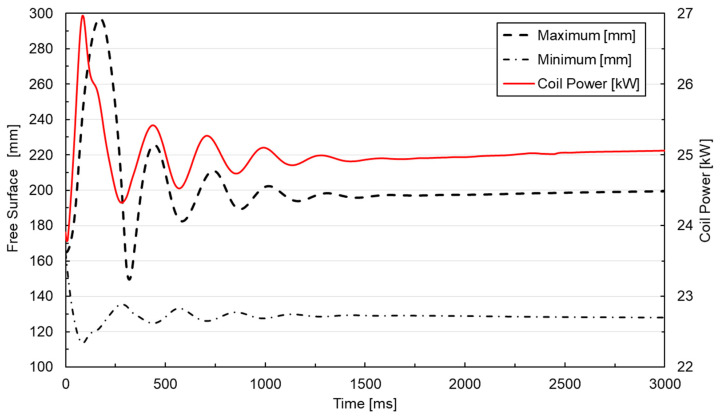
Free surface oscillations and numerical coupling of magnetic fields and fluid dynamics.

Based on the described numerical model, a frequency transient study was conducted. Figure 5 plots the free surface maximum and minimum height evolution among the coil power for the case study simulation of a 10 kg aluminum melt at 25 kW power.

The convergence of the model was verified by demonstrating its ability to reach a stationary solution. The coupled simulation between magnetic fields and fluid dynamics was initially performed for 10 s, but it was found that a couple of seconds were enough to achieve an almost stationary state of the free surface profile. Subsequently, this time was reduced to 3 s for a convergent solution. Regarding numerical stability, an absolute tolerance of 1 × 10^−3^ was applied in the solver for each simulation step to ensure accuracy and robust convergence.

The time step size was similarly chosen to ensure numerical stability, with its impact on the solution carefully assessed during preliminary testing. Fixing it at 5 ms was enough to ensure convergence and numerical stability, capturing the system’s dynamics while maintaining computational feasibility, as decreasing the time step did not modify the results.

The correct numerical coupling between the melt displacement and the magnetic field can be seen, as the coil power was adjusted according to the melt and the electrically conducting domain movement. The oscillations of the metal geometry during the simulation influenced the original magnetic field, leading to delayed variation in the applied power. This accurate coupling between the physics resulted in an almost stationary state solution after a few seconds of simulation. However, the initial fast oscillations were not the actual behavior observed in the melting trials. These oscillations resulted from the instantaneous power application, not reflecting the actual behavior of the power generator, which increases power progressively until resonance conditions are reached. Therefore, a more realistic power application condition is achieved by incorporating a smoothing function with a ramp-up condition, facilitating numerical convergence.

#### 4.1.2. Mesh Independence Analysis

The influence of mesh discretization on the numerical results was evaluated by refining the mesh by progressively reducing the maximum element size and increasing the number of finite elements. The mesh quality was assessed using the skewness mesh metric, quantifying the distortion of an element, comparing its actual geometry to an ideal reference shape, and penalizing elements with large or small angles compared to an ideal equilateral triangle. The same meshing scheme for both interface tracking methods, LS and ALE, was tested to have comparable results.

As a case study, a melting trial of 10 kg and 25 kW was considered, and essential numerical indicators were evaluated to compare the mesh influence on the results. For that, the averaged magnetic flux density ($B_{avg})$, averaged Lorentz force $\left(F_{L\_avg}\right)$ melt velocity ($u_{avg}$), and maximum melt height ($h_{max})$ were assessed inside the multiphase domains (Table 4). A division was made for the interface tracking method, as the maximum melt height and the required computation time differ. Simulations were executed on an Intel(R) Core(TM) CPU i9-10940X node with 64 GB of RAM.

In the case of magnetic flux, precise consistency was achieved, and the results hardly varied due to the small size of the elements capturing the length of the skin depth. The gradual increments for all cases show a slight average Lorentz force increment. A progressive velocity increase was observed in the flow velocities case, probably related to local effects due to reduced mesh elements in the near-wall region, leading to higher velocity peaks. Similarly, there are slight variations between the two interface tracking methods due to numerical diffusion on the free surface interface and the treatment of boundary conditions. The same was true for the maximum melt height, with consistent results for the two methods. For all the evaluated numerical result indicators, the relative error concerning the previous mesh case is below 1%, except for the averaged flow being slightly higher, thus indicating that they are repeatable. It is worth mentioning that the LS interface tracking method requires between 3.5 and almost 4 times more time to complete the simulation, with higher differences for smaller mesh sizes. In order to find a balance between the simulation time requirement and precision, a fine mesh was selected for the posterior simulations.

### 4.2. Magnetic Field Verification

**Figure 6:**
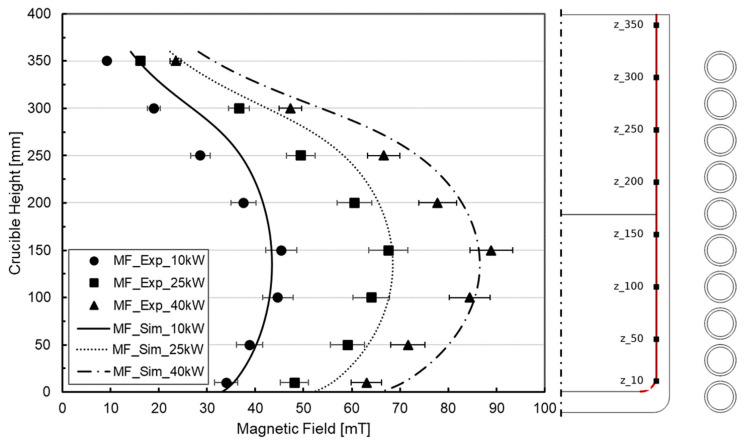
Comparison of magnetic field for simulation results and experimental measurement.

First, the accuracy of the magnetic submodel was studied by comparing the magnetic field generated by the coil with the experimentally measured field. For the simulations inside the crucible, a void domain corresponding to the air was included, while for coil electrical variables, the values for 10 kW, 25 kW, and 40 kW were incorporated, computing the magnetic field in the frequency domain. In the inner contour of the crucible, the numerical magnetic field intensity was extracted and presented, along with the gaussmeter measurement (Figure 6).

The maximum magnitude of the generated magnetic flux density was in the order of 0.1 T, a typical value for this type of electrometallurgical melting technology. The magnetic field profile was appropriately reproduced, and the deviation was between 9% and 11% in average values. It has to be noted that the significant difference is in the top part of the crucible, improving the correlation in the bottom-middle zone where the liquid metal was placed. Here, the deviation is reduced, confirming the validity of the coil modeling procedure.

This overestimation is consistent across all cases, thus resulting in a higher Lorentz force vector and melt velocities, so that higher surface deformations are expected. However, there should be a proportional increase in the relative height of surface deformations for all melting cases considered and for both the ALE and LS free surface tracking methods.

### 4.3. Magnetic–Fluid Dynamic Verification

Once the consistency of the magnetic field was verified, the coupled magnetic field and fluid dynamic simulation were performed for both free surface tracking numerical methods. The experiment was reproduced by parametrizing the melt filling inside the crucible and adapting the electrical coil according to data from Table 3. The material properties of the crucible were switched to aluminum, and the domains were discretized using the same mesh size.

#### 4.3.1. LS Interface Tracking Method

**Figure 7:**
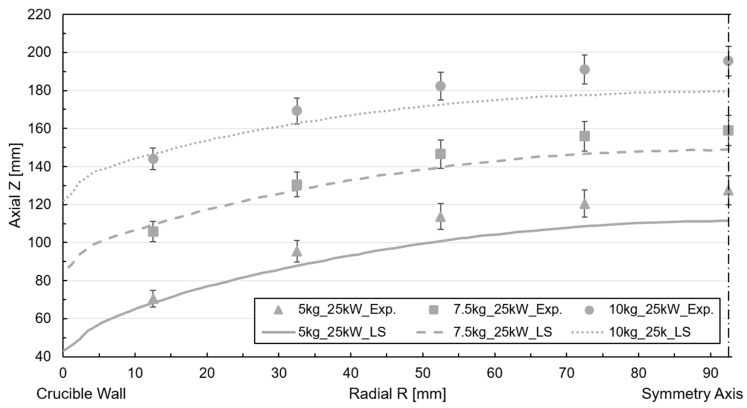
Free surface profile simulation and experimental correlation for LS tracking at 25 kW power.

**Figure 8:**
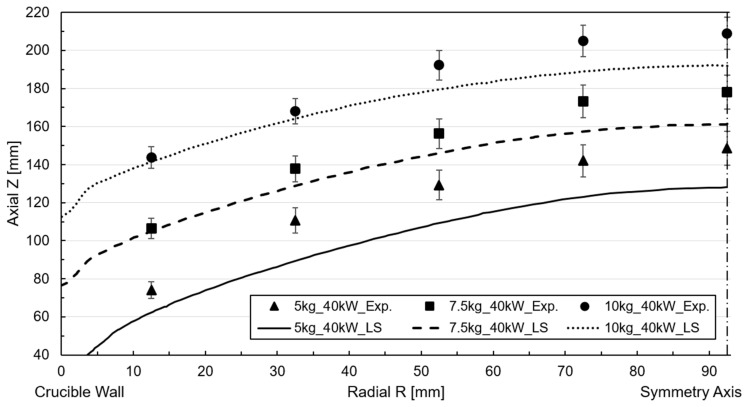
Free surface profile simulation and experimental correlation for LS tracking at 40 kW power.

Free surface tracking using the LS method and simulations were conducted for the six cases mentioned above. Figure 7 and Figure 8 depict the correlation of the free surface by comparing the free surface profile with the experimental measurements for 25 kW and 40 kW power for the iso-surface ϕ=0.5.

**Figure 9:**
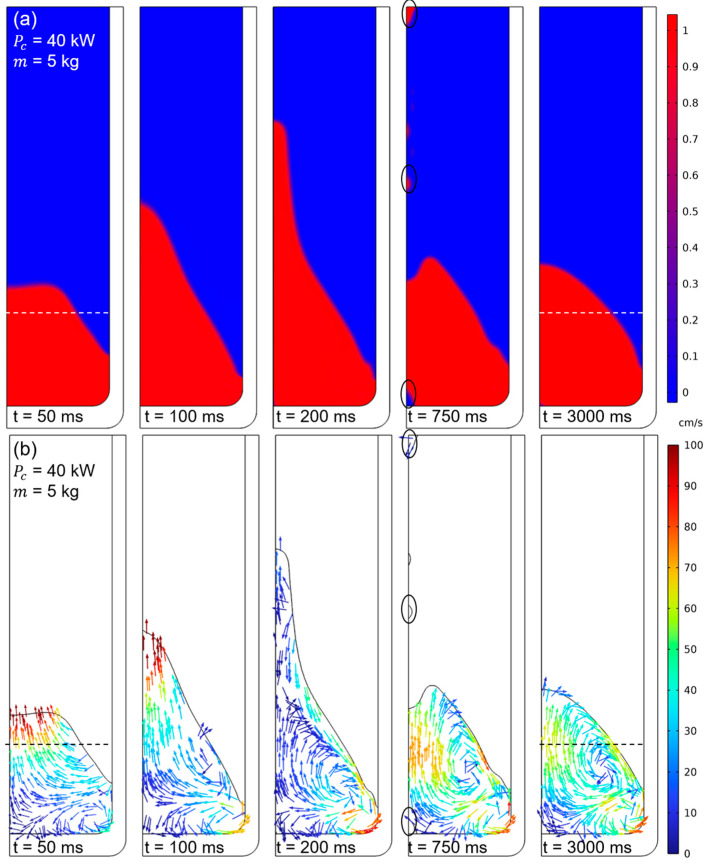
LS simulation results for time steps of interest. (**a**) Multiphase flow. (**b**) Velocity field.

The numerical deformation in the 25 kW power case is slightly lower than the experimental value, with a discrepancy of approximately 15–20 mm across all cases, especially in the melt center reference probe. Concerning metal height, the deviation ranges between 7.5% and 10%. Nevertheless, it is accurate to claim that the profile and height are reproduced satisfactorily. The method is suitable for this type of application, where inertia dominates flow displacement. In the case of a filling volume of less than 5 kg, the divergence is higher due to the increased magnetic field concentration. A comparable arrangement is evident in the 40 kW power case, though the discrepancy with the experimental measurement is more pronounced than in the preceding instance, incrementing the deviation to 15% for the 5 kg mass filling case. An examination of the free surface dynamics for the 40 kW and 5 kg mass scenario, as illustrated in Figure 9a, depicts the evolution of the metal, delineating the metal (red) and air (blue) phases along with the width of the interface at various steps in time during the simulation.

The interphase between the two phases is narrow, reducing the interphase’s numerical diffusion. As can be observed with the immediate application of power, a discernible displacement of the metal is evident. Despite the incorporation of a ramp function for the application of power to mitigate fluctuations, this phenomenon persists. This phenomenon was not observed during the melting process as the power increased. The deformation is so significant that it results in a topological change, mixing the metal and air phases. This is marked with a circle corresponding to 750 ms. Finally, at 3 s, the free surface attains a convex contour profile at the free surface.

Regarding the velocity field (Figure 9b), it is evident that the Lorentz force generates significant momentum, driving the metal upwards. By 100 ms, the formation of two vortices is observed. These vortices gradually conform to the geometry of the metal’s free surface as the system progresses toward stabilization. At equilibrium, two well-defined recirculation patterns emerge. The upper vortex exhibits the highest metal velocities, particularly in the region where the two vortices collide and near the wall adjacent to the lower vortex. Notably, the lower vortex experiences minimal growth due to the combination of high magnetic field strength and the low mass of the liquid metal. This limited growth is further constrained by metallostatic or hydrostatic pressure, which restricts the vortex’s expansion and allows the upper vortex to dominate. The velocity magnitudes range from 10 cm/s to 100 cm/s, consistent with typical values observed in similar applications.

#### 4.3.2. ALE Interface Tracking Method

**Figure 10:**
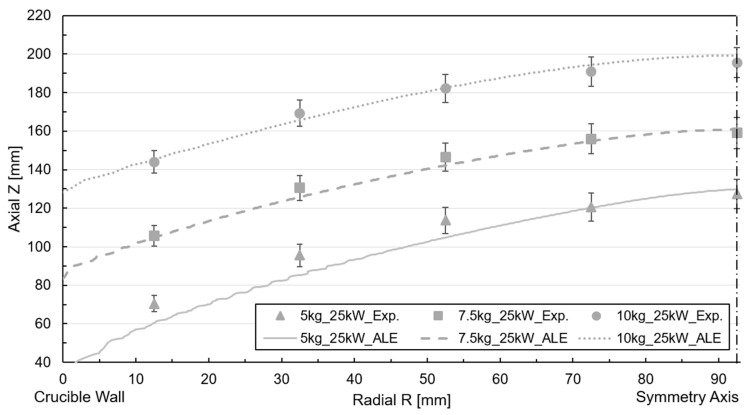
Free surface profile simulation and experimental correlation for ALE at 25 kW power.

**Figure 11:**
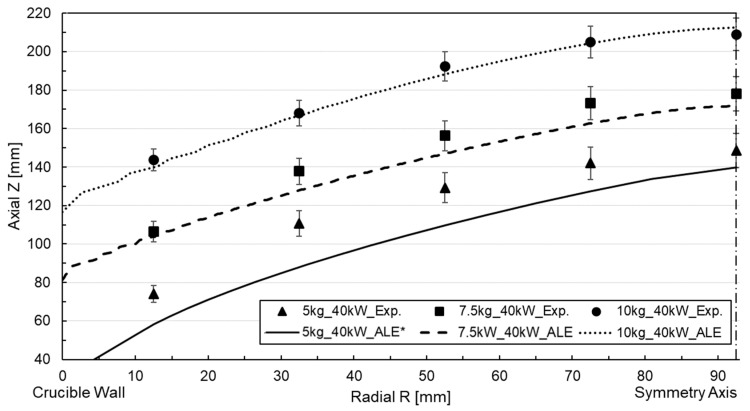
Free surface profile simulation and experimental correlation for ALE at 40 kW power.

For the moving mesh approach, mesh displacement was enabled, and frequency-transitory simulation was carried out with a fixed time step of 5 ms, while the crucible mass-filling and power were parametrized to account for all cases. Regarding the correlation of the magnetic–fluid-dynamic model with the ALE surface tracking method, the deformed mesh was saved once the transient simulation concluded and the surface profile reached an almost quasi-stationary state. The radial and axial coordinates of the mesh elements corresponding to the interface were extracted and plotted alongside the experimental measurements obtained from the height probes. Figure 10 and Figure 11 represent the free surface profile and the height measured by the experimental probes for an input power of 25 kW and 40 kW.

The numerical results for the 25 kW power case effectively captured the curvature profile formed by the point coordinate measurements, exhibiting close replication. However, a slight discrepancy is observed in the near-wall region, which remained consistent across all filling cases. The relative discrepancy concerning the melt height approximates 5% for the near-wall reference probe. This underestimation could be attributed to surface instabilities caused by the low-frequency melt oscillations detected in the experiment. The RANS-based turbulence models utilized in the simulation cannot account for these fluctuations due to their flow-averaging nature [27]. The accuracy of the numerical correlation decreased slightly for the higher deformations associated with the 40 kW power case (Figure 11). In this instance, the simulation yielded a lower height than the experimental measurements, even if it captures the profile’s curvature, resulting in an approximate error of 10 mm for the worst-case scenario.

**Figure 12:**
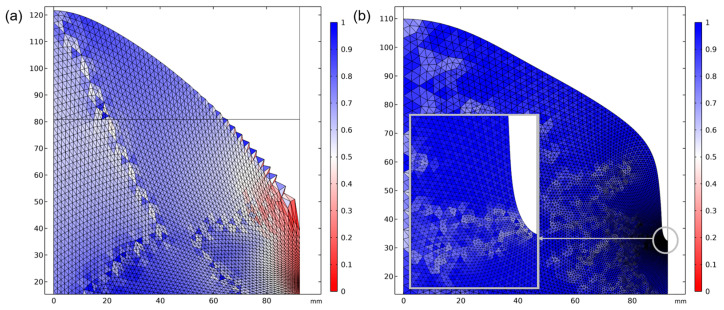
(**a**) Mesh skewness for 5 kg and 25 kW. (**b**) Mesh refinement and near-wall curvature detail.

For the 5 kg filling case, there was an increase in surface profile roughness in the vicinity of the wall, especially between the wall and the first measurement reference. In Figure 12a, the degree of mesh shape distortion, or skewness, is plotted, and it can be seen how mesh elements near the wall and in the interphase between the air and fluid domains experience high distortion.

Conversely, the simulation did not converge sufficiently for the fully coupled simulation at a 5 kg filling level and 40 kW power input. A convergent result could be achieved by eliminating the turbulence variables and considering the flow to be laminar, as indicated by asterisk in Figure 11. However, the deviation compared to the experimental measurement is notable, and the surface convex contour is less profound than for simulations accounting for turbulence. The low filling level and high induced current generated a substantial magnetic force on the free surface border, which resulted in pronounced mesh element stretching and distortion, leading to mesh element rotation.

Two potential solutions were considered: adaptive meshing and remeshing. Reducing the mesh size during adaptive meshing increased element distortion and compromised stability and convergence, causing premature termination. The remeshing method uses moving mesh control to address numerical instability from mesh deformation. Mesh nodes were updated iteratively with a mapping function to account for flow motion until convergence or the simulation ended. A remeshing operation was triggered if mesh element skewness dropped below 0.2. However, the numerical solver failed to converge in the case of high deformation, even after reducing the time step during initialization. The curved shape of the metal made it problematic to initialize the turbulence variable and velocity flow field (Figure 12b).

### 4.4. Free Surface Tracking Methods’ Accuracy and Correlation

To assess the precision of the model and the capabilities of both surface tracking methods, the numerical and experimental results are summarized in Table 5, where the maximum and minimum melt height for both the experimental and simulation models are resumed. Both models demonstrate relatively high precision; however, for the maximum melt height, deviations range from 15 mm to 20 mm for the LS method and slightly lower, at 5 mm to 10 mm, for the ALE method, corresponding to less than 10% in relative terms based on the melt height. For the minimum melt height, the LS method showed slightly better performance in the case of a 5 kg mass filling and 40 kW power input. Nevertheless, the results remain consistent across cases, demonstrating the reliability of both methods.

The data on the minimum melt height further enhanced the results’ reliability by identifying the wetting angle between the crucible wall and the metal. The angle was parametrized to match the free surface profile better. Experimental determinations and simulation identified a wetting angle of 120° as providing the best fit for the measurements.

Two sources of deviation were identified. On the one hand, the potential overestimation of the magnetic field led to higher Lorentz forces, flow velocities, and consequent free surface height. On the other hand, inaccuracies in the measurements obtained from the contact probes may also have contributed. The surface profile was characterized based on the wetting and sticking behavior of the aluminum melt on the rods. Thus, free surface oscillations and damping effects may introduce uncertainties, as the free surface shape is not entirely fixed or stable. Furthermore, the insertion of rods into the metal alters the crucible’s fill volume and influences both flux velocity and oscillations. Ultimately, the experimental trials led to a possible cumulative error from magnetic field measurements, the measuring equipment, and free surface profile heights, contributing to the uncertainty.

One of the principal challenges in validating such models is the limited availability of detailed experimental data to validate induction melting modes. Most studies match their models against the maximum melt height of the free surface, while comprehensive free surface profiles, particularly near the crucible wall, are not measured. This is one of the reasons for the experimental measurements that were performed in this study. In order to double-verify the model, a review of the literature identified three references that provide measurements of the free surface profile with the fixed mesh method [7,9,28].

The proposed ALE free surface tracking method was tested against the experimental measurements reported in these studies. The ALE model was carefully adapted to replicate the reference induction melting furnace geometry, coil configuration, and operating parameters to ensure a valid comparison. The results demonstrated a strong correlation between the simulation and experimental data across all cases. The ALE method accurately reproduced the maximum melt height of the free surface. However, some deviation was observed in the minimum melt height, where higher melt compression was localized in a similar trend to the measurement in this study.

This can be attributed to uncertainties in the actual coil geometry and electrical parameter specifications, which significantly influence the induced currents, electromagnetic forces, and subsequent free surface deformation and the lack of sufficient experimental data near the crucible wall.

These findings highlight the advantages of the ALE method, which outperforms LS by achieving higher accuracy in modeling free surface profiles under realistic operating conditions. Additionally, the ALE method demonstrated shorter computation times, further supporting its applicability to complex induction melting systems.

### 4.5. Metal Stirring and Flow Analysis

The standard k−ε model is designed for high Reynolds number flows above 10^5^ where the flow is fully developed or in free stream conditions. The model struggles with adverse pressure gradients, often overestimating shear stress and delaying flow separation, leading to underestimation of recirculating zones and rotating flows [26].

A near-wall modeling approach was applied to address these limitations, integrating turbulence equations through the buffer and viscous sublayer to the wall and solving the flow velocity field. The AKN low Reynolds number (LRN) k−ε turbulence model was adopted for its ability to predict flow separation and attachment. Nonlinear damping factors are incorporated to improve turbulence transport equations. For the no-slip wall condition, the velocity was set to zero (u=0), and the wall boundary conditions for turbulent kinetic energy (k=0) and dissipation rate ($\varepsilon =\frac{2\mu }{\rho }k/l_{w}^{2}$) were applied [29].

**Figure 13:**
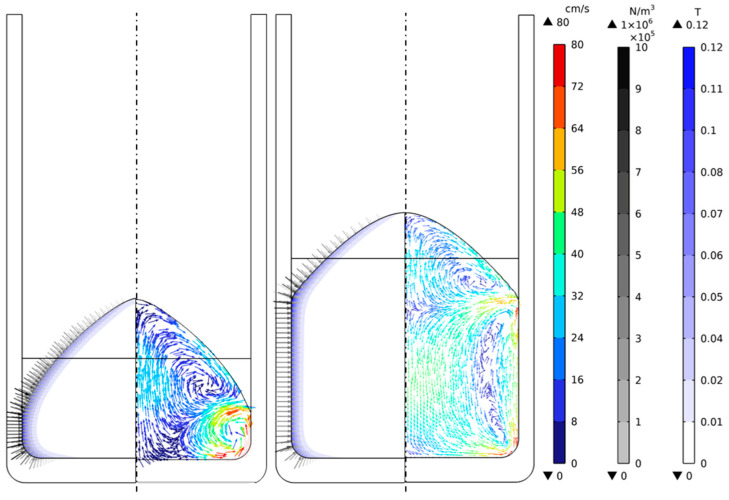
Magnetic field, Lorentz force, and flow field for 5 kg and 10 kg at 25 kW power.

The requirement for a finer mesh increased the demand for mesh quality, leading to additional mesh refinement to ensure the proper distance of the center of the initial mesh element from the wall. With these adaptations and fixing the free surface, a frequency-stationary study was computed using the magnetic, flow fields, and turbulence variables. As a representative case, the mass filling of 5 and 10 kg and 25 kW is selected to plot the magnetic field, Lorentz force vectors, flow field, and free surface profile (Figure 13).

Regarding the electromagnetic or Lorentz force, whose vectors are plotted, their maximum values are directed axially toward the melt’s center axis. It is concentrated in the region near the wall at the ends when the field comes into contact with the conducting medium. Furthermore, as far as the Lorentz force is concerned, it is observed how it is focused on the outer area of the metal due to the film effect, which is around 4.9–5 mm for the case of liquid aluminum at frequencies of the order of 3 kHz. The magnetic field strength for all cases in the 25 kW power case is around 0.12 T, and the maximum Lorentz force is 1 × 10^6^ N/m, with the highest peak being for the 10 kg filling. These forces also cause an increase in the flux velocity in the crucible wall area, related to a concentration of the forces at the end of the free surface closest to the wall.

For the 10 kg filling case, the averaged flow field indicates the formation of two primary/principal eddies with a stirring effect in the opposite direction, leading to the stirring of the melt. This stirring compresses the vortices against each other, accelerating the flow in the central region and the acceleration in the crucible near the wall region due to the concentration of the magnetic field and the collision of the two vortices, making the stream flow upwards. However, for the 5 kg case, a principal vortex can be found on the crucible curvature radius. Here, the velocity of the flow is rapid, while the free surface decays notably. The low mass filling and high magnetic pressure do not lead to the complete formation of the second vortex due to a higher field concentration at a smaller height and less space for flow development. Even if it is not plotted, in the 7.5 kg filling case, two primary vortices are formed with a recirculatory pattern similar to the 10 kg filling case, with flow velocities in the same range.

The magnitude of the flow field is in cm/s, which is a typical value for this type of induction melting application—reaching a maximum melt velocity of 80 cm/s for the 5 kg case, while for 10 kg, it is 65 cm/s. The velocity of the lowest mass filling is higher, probably due to the accelerating effect of the concave geometry of the bottom side of the crucible and the concentration of the magnetic force in that area. In the center of the vortex, the flow is almost steady, and in the region between the two vortexes and symmetry axes, r = 0 at the base and top of the crucible.

Although not depicted for the 40 kW case, the flux distribution is practically similar to the peculiarity that the magnitude of the flux velocity exceeds 80 cm/s for the 10 kg filling and 100 cm/s for the 5 kg filling. At the same time, the induced magnetic field exceeds 0.15 T and has a maximum Lorentz force of 1.5 × 10^6^ N/m.

**Figure 14:**
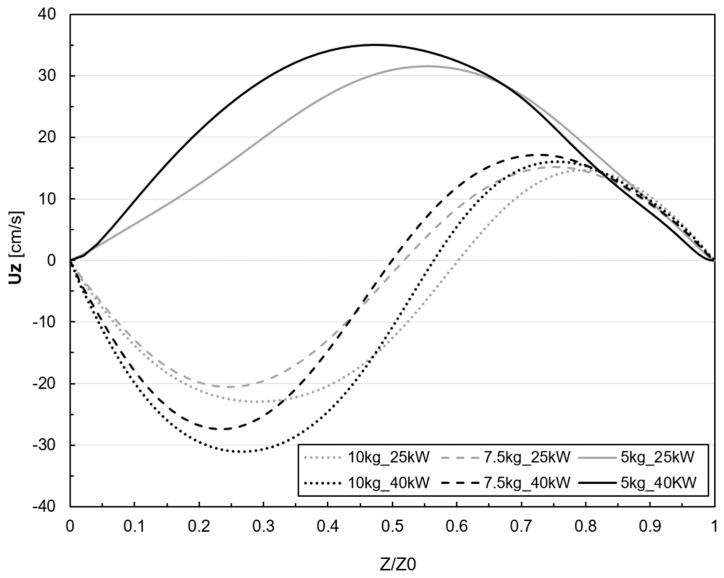
Axial velocity ($u_{z}$) in r=0 axis for the case studies.

In order to provide a comparative sample, the axial velocity component ($u_{z}$) is plotted for the r = 0 axis, which is situated directly in the center of the axis, for the six simulation cases (Figure 14), with the height of the metal on the z-axis normalized.

The flow’s axial velocity vector ($u_{z}$) reveals the presence of two distinct peaks, one positive and one negative, indicating opposite flow rotation directions. These peaks correspond to the narrowing of the flow due to stirring, which leads to acceleration when the two streams come into contact. The velocity becomes zero in the zone where the two eddies are in contact, between 0.5 and 0.6 of the normalized Z0 height. For the 5 kg case, only the positive component can be observed, as the second vortex is localized in the wall region and much smaller than the superior, dominant one. For higher powers, the peak velocity is almost 10 cm/s larger, while the collision of the vortexes is centered.

An increase in axial velocity at the melt center generates a corresponding rise in thrust force, intensifying the free surface’s deformation. This effect is particularly evident in the case of the 5 kg filling, where the velocity field exhibits exclusively positive axial components, resulting in a more significant free surface displacement, as summarized in Table 5. For the 7.5 kg and 10 kg cases, there is a positive and negative axial velocity component, slightly reducing the free surface maximum height.

For all the cases, the average turbulence number of the flow ranges between 1.5 × 10^4^ and 3 × 10^4^, being in transition between laminar and fully turbulent; thus, the LRN turbulence model adaptation is a legitimate solution for a more precise melt flow simulation.

## 5. Conclusions

A comparison of moving and fixed mesh methods for simulating the free surface profile for induction is made. The precision of the model is confirmed by experimental measurement correlation for the free surface contour and magnetic field. Similarly, robustness is assured with sensibility analysis for key numerical result variables.

- Topological changes occurred for the LS fixed mesh method, mixing metal and air bubbles inside melt and metal flow separation, which is not the actual behavior seen during the melting trials. Even though mass conservation is ensured and the results are favorable, they could not reproduce the free surface shape in detail, leading to deviation compared to the experiment, especially for high power levels.
- On the contrary, the ALE method avoids topological changes, facilitates convergence, and requires less computation time than the LS method, thus increasing accuracy. The relative error for the ALE method is smaller than for LS. The absolute height of the melt is achieved while the deviation in the near-wall region increments.
- Getting a satisfactory and convergent result for 5 kg and 40 kW power was not possible. A convergent solution was achieved by assuming the flow to be laminar but forgoing precision and emphasizing the importance of including the turbulence transport variables. Thus, the ALE method is more favorable for not too large free deformation cases that require rapid and precise solutions.
- The LRN k−ε turbulence model adaptation is suitable, as it can resolve the flow field up to the wall, and a recirculation pattern is observed. Two dominant vortices in opposite directions, with a magnitude of about 60 cm/s to 80 cm/s are formed, similar to these electrometallurgical processes.

The ALE method has been identified as the best solution regarding accuracy and simulation time for the free surface tracking method for applications where magnetic field–fluid dynamic coupling is considered. While focused on induction melting, it can be extended to other industrial processes, such as cold crucible induction melting (CCIM), levitation melting, and continuous casting of steels. These processes involve varying degrees of surface deformation, where the model’s capabilities in free surface tracking offer significant advantages. While larger deformations in levitation melting may require further refinements to ensure convergence, the model is particularly well suited for more stable processes like continuous casting. Additionally, the computational efficiency of the 2D axisymmetric geometry and enhanced ALE approach facilitate its scalability to industrial-sized applications.

## Figures and Tables

**Table 1:** Resume of principal materials parameters and geometrical dimensions.

Material Parameters			Geometrical Dimensions [mm]			
	Aluminum	Air	Coil		Crucible	
Density ρ [kg/m^3^]	2300	1.204	Turn outer Ø	30	Inner Ø	185
Viscosity μ [kg/(m·s)]	1.29 × 10^−3^	1.81 × 10^−5^	Turn inner Ø	25	Outer Ø	210
Electrical conductivity σ [S/m]	3.6 × 10^6^	0	Coil total height	345	Inner height	360
Surface tension γ [N/m]	0.85	-	Coil inner Ø	300	Outer height	380
$\mathrm{Wetting}\;\mathrm{Angle}\;\theta_{w}$ [deg]	120	-	Nº Turns	10	Inner radius	15

**Table 2:** Boundary conditions for the numerical model.

Boundary	Physical Submodel			
	Flow Field	Turbulence	LS	ALE
Wall	u·n=0 $K\cdot n=-\rho \frac{u_{\tau }}{u^{+}}u_{tang}$	∇k·n=0 $\nabla \varepsilon \cdot n=\frac{Ck_{\mu }^{2}}{k_{v}\delta_{w}^{+}\mu }$	$k_{nt}\cdot n=\gamma \delta \left(n\cdot n_{wall}-\cos\left(\theta_{w}\right)\right)\;\;\;\;\;\;\;\;\;\;\;\;\;\;\;\;\;\;\;\;\;\;\;\;\;\;\;\;\;-\rho \frac{u_{\tau }}{u^{+}}u_{tang}$	$d_{r}=0$ $u_{tang}=u-\left(u\cdot n\right)n$
Fluid–Fluid Interface	$u_{1}=u_{2}$ $n_{i}\cdot K_{2}=n_{i}\cdot K_{1}+F_{ST}$	$k_{1}=k_{2}$ $\varepsilon_{1}=\varepsilon_{2}$	ϕ=0.5 $\frac{\partial \phi }{\partial t}+u\cdot \nabla \phi =0.5$	$u_{mesh}\cdot n_{i}=u\cdot n_{i}$
Open Boundary	K·n=0	∇k·n=0 ∇ε·n=0	n·ϕ=0	$d_{r}=0$ $d_{z}=0$

**Table 3:** List of measured electrical variables for free surface characterization tests.

Mass [kg]	$P_{c}$ [kW]	$f_{c}$ [Hz]	$I_{c}$ [A]	$V_{c}$ [V]	φ [deg]
5	25	2816	511	538	84.9
	40	2864	641	694	84.7
7.5	25	2820	514	541	84.8
	40	2908	614	644	84.3
10	25	2916	483	478	83.8
	40	2980	668	694	85.0

**Table 4:** Results of mesh independence analysis.

Meshing				
	Coarse	Normal	Fine	Refined
Nº of Elements	3.2 × 10^4^	4.7 × 10^4^	1.1 × 10^5^	1.4 × 10^5^
Max Length [mm]	10	7.5	3.5	2
Avg. Skewness	0.82	0.83	0.86	0.88
Numerical Results				
$B_{avg}\;$ [mT]	27.24	27.07	27.03	26.91
$F_{L\_avg}\;$ [N/m^3]	3.98 × 10^4^	4.01 × 10^4^	4.03 × 10^4^	4.05 × 10^4^
LS Interface Tracking				
Computation time	19 min 29 s	27 min 21 s	1 h 12 min 29 s	2 h 18 min 23 s
$h_{max}$ [mm]	183.02	183.65	184.32	184.48
$u_{avg}\;$ [cm/s]	30.39	30.73	35.78	37.13
ALE Interface Tracking				
Computation time	5 min 52 s	7 min 34 s	18 min 33 s	33 min 45 s
$h_{max}$ [mm]	193.32	194.43	195.45	195.74
$u_{avg}\;$ [cm/s]	32.96	34.83	37.99	40.54

**Table 5:** Simulation results and experimental measurements comparison.

Power [kW]	Mass [kg]	Max. Free Surface [mm] (r = 92. 5 mm)			Min. Free Surface [mm] (r = 12.5 mm)			Total Free Surface Deformation [mm]	
		Exp.	Sim. ALE	Sim. LS	Exp.	Sim. ALE	Sim. LS	Sim. ALE	Sim. LS
25	5	127	128	112	71	61	69	93	68
	7.5	159	161	149	106	106	109	77	64
	10	195	199	180	144	143	145	71	57
40	5	148	140	128	74	59	63	106	94
	7.5	178	171	161	107	102	104	96	85
	10	209	213	192	144	139	143	91	79

## Data Availability

The data presented in this study are available on request from the corresponding author.
